# Feasibility and attractiveness of indication value-based pricing in key EU countries

**DOI:** 10.3402/jmahp.v4.30970

**Published:** 2016-05-10

**Authors:** Mathias Flume, Marc Bardou, Stefano Capri, Oriol Sola-Morales, David Cunningham, Lars-Ake Levin, Nicolas Touchot, On behalf of Payers’ Insight

**Affiliations:** 1 Kassenärztliche Vereinigung Westfalen-Lippe, Dortmund, Germany; 2 CIC INSERM 1432, CHU du Bocage, Dijon, France; 3 School of Economics and Management, Cattaneo–LIUC University, Castellanza, Italy; 4 Health Innovation Technology Transfer, Barcelona, Spain; 5 NHS South East Commissioning Support Unit, London, UK; 6 Department of Medical and Health Sciences, Linköping University, Linköping, Sweden; 7 groupH, London, UK

**Keywords:** indication based pricing, drug value, pricing and reimbursement

## Abstract

Indication value-based pricing (IBP) has been proposed in the United States as a tool to capture the differential value of drugs across indications or patient groups and is in the early phases of implementation. In Europe, no major country has experimented with IBP or is seriously discussing its use. We assessed how the reimbursement and pricing environment allows for IBP in seven European countries, evaluating both incentives and hurdles. In price setting countries such as France and Germany, the Health Technology Assessment and pricing process already accounts for differences of value across indications. In countries where differential value drives coverage decisions such as the United Kingdom and Sweden, IBP is likely to be used, at least partially, but not in the short-term. Italy is already achieving some form of differential value through managed entry agreements, whereas in Spain the electronic prescription system provides the infrastructure necessary for IBP but other hurdles exist.

Targeted medicines are frequently developed for different diseases/indications with common underlying biological mechanisms. For example, in oncology a specific drug may be developed for multiple cancer types and within each cancer for several lines of therapy. The product benefit and therefore its ‘value’ is almost certain to vary by indication and by treatment line. For example, Xarelto is indicated for stroke prevention in atrial fibrillation, for the treatment and prevention of deep vein thrombosis and pulmonary embolism, and for primary prevention of Venous Thromboembolism (VTE) in orthopaedic surgery. In today's reimbursement environment, the price of the product will be uniform across usages, the difference being restricted to dosing. Although some market access schemes based on ‘payment by results’ can be implemented, such as Roche with Avastin in Germany and Italy (1), those do not allow payers to fully capture the difference in value found across indications.

In the United States, a discussion is emerging around indication-based pricing (IBP). Frequent pricing critic Peter Bach recently suggested that paying by indication could save money in cancer (2) using the example of cetuximab, which is much less effective in advanced head and neck cancer (estimated value-based price: $470) compared with colorectal cancer (estimated value-based price: $10,320). More recently, Express Scripts (3), a leading full-service pharmacy benefit management company, has been trying to peg the cost of cancer drugs to their clinical benefits. Pharma companies can opt into the IBP program to ensure their treatments are covered across indications. If they decline, therapies with variable success might only be covered in the indications where they have established superiority, but not in others, provided suitable alternativesare available. Express Script intends to roll out the approach for cancer therapies first, with anti-inflammatory treatments to follow (3).

Ultimately, products may never be developed in an indication for which they could represent a major therapeutic advance, simply because this would lead to lower price than that achieved or achievable in other indications. Therefore, the product will likely be used off-label at a much higher price than it would deserve. There are three policy options:

1. Prohibit off-label use and prevent patients from accessing a last-resort effective therapy
2. Accept off-label use and overpay, leading to an unjustified burden for health insurance
3. Develop policies to allow IBP

Although current policies may not open for IBP in most countries, it is worth reviewing the potential way forward for such policy.

Whether IBP will become a reality in the United States, and if so in which indications (oncology only or beyond), is still debated. In this article, we explore and discuss whether a similar policy would be feasible in key European countries and, if possible, how it may create value in selected European health systems.

## Germany

Today there is no IBP with different prices per indication implemented. This situation leads to discussions in cases where different indications receive different levels of additional benefit. Currently, the head organization of the sickness funds suggests an IBP concept to focus the reimbursement on subindications with high additional benefit. This idea is not widely supported by the sickness funds in Germany today. It is complex to implement and furthermore against the current policy to make drugs available for indications without additional benefits (4).

In Germany, all new drugs are regulated by the Arzneimittelmarkt-Neuordnungsgesetz (AMNOG) framework (5, 6) as far as they are considered as new entity launched after 2011, with a mandatory price negotiation leading to one price across indications. For each indication, and subgroup when relevant, a comparator is set by the Gemeinsame bundesausschuss (GBA), and the extent of additional benefit and the size of the target population are assessed. Those parameters form the base for a single price negotiation. This process is repeated for novel indications. Thus, the different value of a drug across indications or subgroups is already embedded in the pricing system. Ultimately, the manufacturer will receive a single price representing a volume-weighted average price per indication. Therefore, an IBP scheme would likely fail to generate significant savings for the sickness funds.

Indications without additional benefit usually fail to generate price premiums over the current standard of care. As a result, in many cases companies refuse to register a new indication to avoid price erosion. Off-label use is seldom considered, as the physician may have to pay for the drug in case of unauthorized prescription. Thus, instead of considering IBP, companies may simply choose to restrict indications to the ones with the highest price potential.

Payers receive only ICD-10 data, lacking the granularity required to implement IBP. Setting up a more refined data collection process without clear financial incentives is unlikely. In addition, the high level of data protection in Germany makes IBP difficult. For example, currently the indication for which a drug is used is not noted on the prescription and is not communicated to the pharmacist or the sickness funds.

At present, price differentiation by indication is possible only by individual contracts with regional sickness funds providing discounts on top of the AMNOG-negotiated price. However, this is contrary to the philosophy of the German pricing system and would not lead to IBP, since the highest value price would never be reached. In the future, it could be possible to consider a federal price based on the highest value indication with confidential discounts agreed on for lower value indications and applied at the federal (national) level. This scheme may be attractive for the industry to maintain a high German price for international referencing, but would require a complete change in the German Social Code book (5, 6), which is unlikely given the lack of benefits for all other stakeholders.

Thus, we believe that IBP is unlikely to occur in Germany within the foreseeable future. The current approach, which already takes into account high and low value indications, is perceived to be the best way to ensure affordability of novel therapies and is likely to persist.

## France

There is little discussion on IBP in France, one reason being the lack of incentives. As in Germany, the current system already reflects value across indications, and the implementation of IBP is unlikely to result in significant savings for the National Social Security System.

When a product has several indications, whether relating to different conditions or subgroups of patients within the same condition, clinical benefit and incremental clinical benefit (ASMR) ratings are granted for each indication separately (7, 8).

In addition, clinical comparators and target population size are also defined for each indication. The pricing committee then defines an average price that already represents the value across indications weighted by the expected volume. The manufacturer is then requested to perform a real-life study to document the volume weight anticipated at the time of pricing was appropriate. If the weight foreseen at the time of negotiation does not match the actual volume weight, the price is then revised.

This approach provides a key element for IBP: the evaluation of a specific value per indication. However, it also eliminates all incentives for IBP.

In addition, when a product is granted a high ASMR (I to III), the marketer is entitled to the EU reference pricing for the first 5 years of marketing. In the case of an indication extension before the 5 years, the company loses the right to the EU reference price, regardless of what the new ASMR is, even I. This is a disincentive for line extension or new indication during the first 5 years.

For expensive drugs usually charged on top of the Diagnosis-related group (DRG), for in-hospital use (*liste en sus*) a ‘proper use contract’ is required to ensure that the drug is prescribed for the appropriate indication and line of therapy (9). Failure to complete that document leads to no or partial reimbursement to the hospital. This mechanism may in theory also be used to define a different reimbursement level per indication for in-patient use. However, this does apply only to a minority of products provided by the hospital, usually on the top of DRG, and no mechanism exists for community products. Moreover, university hospitals are not actually controlled or sanctioned for off-label use.

Another limitation is the inability to monitor prescriptions by indication outside of the hospital, because it is forbidden to write the indication on the prescription (10). This would only be possible when a different branded name (e.g., celecoxib marketed for rheumatoid conditions as Celebrex^®^ and familial adenomatous polyposis as Onsenal^®^) or a different formulation (e.g., propranolol that has received an orphan designation as Hemangiol^®^ 3.75 mg/ml for paediatric haemangiomas) is granted. This differential branding is unlikely to happen within the same therapeutic area, such as oncology, where the same physicians often prescribe for multiple cancer types, but could theoretically be implemented across different therapeutic areas, as for example with biotherapies targeting different inflammatory conditions such as inflammatory bowel diseases and rheumatoid arthritis. This could only work if the product were available in different formulations, dosages, or modes of administration. Otherwise healthcare providers might use the cheaper option, as illustrated by the use of Avastin for Age-related Macular degeneration (AMD) through the temporary use recommendation (11).

In the foreseeable future, we do not expect development of IBP, as this would require a deep transformation of the French price-setting process and would bring limited benefits to payers.

## Italy

In Italy, there is no discussion on formal IBP, but net prices (the purchase price for the hospital) already vary across indications for many drugs through managed entry agreements. Italy has 127 registries (12) allowing implementation of three types of managed entry agreements: 1) payment by results – full refund for non-responders based on outcome evaluation; 2) cost-sharing – a partial refund of the first cycles of the therapy for eligible patients; 3) risk-sharing – a partial refund of a few cycles of therapy for patients not responding at the point of re-assessment of the patient. By applying different patterns of managed entry agreements for different indications, Agenzia Italiana del Farmaco (AIFA) *de facto* obtains different net prices for different indications. The most striking case is that of bevacizumab (Avastin), which is currently reimbursed in seven oncology indications and in macular degeneration (13). In colorectal neoplasms, a risk-sharing scheme is applied. In contrast, in the other oncology indications payment by result schemes are in place, but those use different rates of disease progression for different cancers. As a result, net prices vary for each cancer type based on outcomes.

The reimbursement system in Italy results from a negotiation between AIFA and the manufacturer (14). It defines an ex-factory price, with the addition of some mandatory discounts and increasingly with a price cap volume agreement at the national level. When a drug obtains a second or a third indication, with a significant increase in the number of target patients, two approaches can be used: either an ex-factory price reduction or the extension of the undisclosed discount to maintain the same ex-factory price. Therefore, in principle, the ex-factory price is independent of the different therapeutic indications.

However, as already mentioned, net prices already vary across indications through managed entry agreements. Those agreements are usually not confidential and often include pay for performance beyond discounting and price volume agreement.

Each registry allows the purchase and utilization of the drug only if a specific patient record/form is filled, then transmitted to the hospital pharmacist, to AIFA, and to the manufacturer. Therefore, prescription and billing are easily traceable. Data collected through the registries are owned by AIFA. In oncology registries, >80% of entry is achieved within a week (15).

Although data collection is easy to perform, as it is essential for the prescription and the patient's monitoring, this system is time consuming for the clinician and for the hospital pharmacist. There are also extra costs for creating the registry and for the maintenance. As a result, the refunds from patient access schemes are not always captured. In 2012, out of €46.2 million of eligible reimbursement (payback), only €31.2 million (67.5%) was reimbursed (€5 million was not requested by hospitals and €9.9 million was not validated by pharmaceutical companies) (16).

In Italy, drug pricing does not discriminate among different indications for the same drug, but in practice some net prices are already indication-based. Implementing open IBP would require a change in the legal framework and is unlikely in the short to mid-term, but broad use of patient access schemes will continue in oncology and may develop in other indications. However, while net prices depend on the outcome in each indication, their difference does not necessarily represent the real difference in value.

## Spain

While it has been occasionally proposed in closed forums in the past years, IBP remains an academic concept or option in Spain without concrete progress and with few short-term expectations. It has not been openly discussed and has no real advocates, probably because of the legal framework.

Although no legislation impedes IBP, prices in Spain are usually set for an Anatomical Therapeutic Chemical (ATC) code (17) and linked to a single formulation, regardless of the potential for diverse indications.

Specific to Spain within the larger area of the EU is the presence of electronic prescription systems organized at the regional level for ambulatory prescriptions. Although details vary across regions, these systems are a comprehensive health management tool that addresses the entire process involved in pharmaceutical prescribing and dispensing. In theory, this provides a tool for both data collection and billing required for IBP. In most regions electronic prescription systems cover a very large majority of community primary care and specialty prescriptions (18, 19).

However, implementing IBP in Spain still requires facing two main hurdles:

1. Despite the increasing consistency of coding across the Spanish NHS, we are still far from a homogeneous system and most regions do not consolidate their healthcare activities in a timely manner. The divide is even greater when it comes to procedures. DRGs (or similar metrics) are available for most of the territory for *post hoc* analysis, but with significant time lags.
2. Most expensive therapies, even when used in the community, are delivered by hospital pharmacies and purchased out of the hospital budget. This budget is a lump sum calculated on an annual basis from historical trends and estimated future needs. An IBP approach would require a complete transformation of the funding mechanisms and information system for hospital management and more specifically hospital pharmacies.

Some regions have begun using mandatory registration of patients for certain drug indications, or centralized authorization of several high cost drugs, thus creating an environment facilitating future implementation of IBP. At present, the drugs included in such schemes are mostly cancer therapeutics and orphan drugs, but the inclusion does not depend on clear prespecified criteria. Only consistent evidence of the benefits of maintaining and analysing such huge databases despite the increased transaction costs will generate political support and trigger a broad change in policies. In addition, Spanish payers have also expressed concerns about IBP related to DRG creep (claiming for the highest possible cost/price per indication) and to loss of (prescription) control when a treatment can be used in several lines of therapy.

Given those concerns, most stakeholders in the Spanish health system currently prefer the current ATC-based pricing to the perceived implementation complexity and potential for error associated with IBP.

## England

Indication and outcome-based pricing, defined as a graded payment system based upon indications or patient outcomes, is not widely used in the United Kingdom.

In England, NICE will review the incremental cost-effectiveness ratio (ICER) and recommend the drug in all indications when the ICER is below the threshold (20). The current system thus relies on reimbursement decisions at a given price, and those are by nature binary: Drug A might be reimbursed at price £X for indication X, but would receive zero (£0) payment for indication Y. As in other countries, the exception would be drugs with dual branding such as finasteride (prostate disease and hair loss). Therefore, in theory there is no option for differential price per indication, at least for drugs that are reviewed by NICE. However, opportunities may exist to provide discounts that vary across indications through patient access schemes, as highlighted in the Pharmaceutical Price Regulation Scheme 2014 (21), using approaches such as dose-capping, outcomes-based schemes, or stocks supplied at zero cost.

The reimbursement system within the NHS in England is complex. Once an individual drug receives market authorization and, if required, a positive NICE assessment, it can be reimbursed from multiple budgets, including the primary care prescribing budget, hospital-based tariff (payment by results – PbR), PbR-excluded drug budgets (also known as high-cost drugs, such as drugs for HIV or antifungals) (21), and for some oncology medicines the Cancer Drugs Fund (22). It is unlikely that a drug would have indications across all budgets, but there are many examples of drugs where reimbursement is drawn from multiple budgets depending upon the indication, treatment setting, or some other national or local parameter. This situation creates a significant barrier for the implementation of IBP.

However, in oncology the Systemic Anti-Cancer Therapy (SACT) data set covers chemotherapy treatment for all solid and liquid tumours (23). All NHS trusts providing cancer chemotherapy services are required to provide monthly data on use of chemotherapy using SACT. This could provide a tool for the implementation of IBP.

As part of the current national strategy (Five Year Forward View), indication- and outcomes-based commissioning (payment) is being undertaken, within the NHS Right Care programme (24) in multiple sites across England. This covers many aspects of care, for example, all care within diabetes or for individual pathways, as for hip surgery. At present, individual medicines have not been covered using this methodology, but in the author's view it is likely that indication- and outcomes-based pricing for individual drugs will become an important tool for reimbursement, although this will more than likely be in the medium term, rather than short term.

## Sweden

Indication-based pricing, defined as a graded payment system based upon indications or patient outcomes, is not currently being discussed in Sweden.

The Swedish government subsidises medicines and the Dental and Pharmaceutical Benefits Agency (TLV) is the agency responsible for the national reimbursement scheme for prescription pharmaceuticals. The reimbursement decision is based on an ethical platform that builds on three main principles: human value, need and solidarity, and cost-effectiveness (25).

Drugs can receive two main types of subsidies: general or restricted (26). With general reimbursement, a medicine has approved reimbursement for its entire area of use. With restricted reimbursement, the drug is included in the pharmaceutical benefit scheme only for certain indications or population subgroups. The dominant reason for restricting reimbursement is that among several indications, the drug is cost-effective in only one indication and/or for a specific subgroup of patients. Another reason could be that the patients lack treatment options as they are not eligible to available first-line medicine (25).

Traditionally, companies did not negotiate prices in Sweden. Companies used to offer a price, along with a rationale and evidence, and TLV could accept or reject it. If rejected, the company could refile with another price. A recent development in the Swedish reimbursement system is three-party negotiations between TLV, the Swedish Association of Local Authorities and Regions, and the drug company (27). This system has begun to be implemented, but remains an option rather than an obligation.

In the current reimbursement system of prescription drugs, there is no role for IBP more than the mentioned binary variant of restricted reimbursement. When the price of a drug is the same for all indications, the drug's cost-effectiveness varies as the drug usage expands to cover more indications and wider patient groups. With a price fixed, there is an obvious risk of suboptimal drug allocation where cost-effective indications are not reimbursed when the average ICER is considered above the threshold.

To implement IBP, prescriptions have to include the indication or diagnosis, which is lacking today. The billing of outpatient drugs from the pharmacy to the county council would be based on the prescription, implying a risk that the prescriber might choose the lowest-priced indication for all patients in order to minimize drug expenditure. The indication/diagnosis could be controlled for in the administrative systems but would still be managed by the county council.

In the near future with the fast development of the negotiation component in the reimbursement decision process, there will be openings for IBP through, for example, indication-based discounts or indication-based risk-sharing. This is already possible but poorly utilized in the process of national or regional procurement of hospital drugs (28).

## Conclusions

At present both use of and interest in IBP are limited in Europe. Other than Italy, no other country routinely sets prices that differ significantly from indication to indication except for multiple brand products. Even in Italy, where price differences may exist, especially in oncology, the variability in net prices across indications is driven by managed entry agreements, which is not true for IBP. As a result, these differences in price do not necessarily represent differences in value.

In price-setting countries such as Germany and France, the Health Technology Assessment (HTA) process reduces the need for indication-based pricing. Negotiated prices already represent a form of weighted average price, representing the value in various indications. There is therefore little incentive for payers to implement a real IBP approach, as overall expenditure would not be greatly reduced.

In countries relying on ICER, such as the United Kingdom and Sweden, coverage depends on the value driven by the ICER threshold. Therefore, the current system is binary and coverage is the variable, not price. However, IBP could develop, most likely in the mid-term rather than in the short term.

Moving forward, implementation of IBP appears difficult in most European countries covered in this analysis. Looking at the feasibility of implementing IBP across several dimensions – legal, data collection, billing – each country appears to have significant hurdles (Table 1). Germany and Italy would both require changes in the legal price-setting environment, whereas the regional organization of the Spanish health system creates complexity.

**Table 1:** Feasibility and hurdles to indication-based pricing

Country	Legal feasibility	Data collection feasibility	Billing feasibility	Other hurdles
Germany	Not within the current German Social Code book	Limited	Limited	Lack of incentives
France	Yes	Restricted (expensive drugs provided by hospitals)	Restricted (expensive drugs provided by hospitals)	Lack of incentives; physician resistance
Italy	Not with current price-setting by AIFA	Yes, full with registries	Yes, full with registries	Physician resistance; extra cost for registries
Spain	Yes	Yes	Yes	IT limitations; regional fragmentation; indication appropriateness
England	Yes	Yes	Yes	Budget fragmentation
Sweden	Yes	Limited	Hospital	Indication shift

As a result, it is likely that IBP will not develop significantly in the near future. Instead, two mechanisms are likely to continue to be used to account for the difference in value across indications:

1. Continued use of a volume-weighted average price defined through HTA outcome across indications, in France and Germany mostly.
2. Increased or continued use of managed entry agreement in Italy, the United Kingdom, and Sweden, at least for expensive products such as cancer therapies. In these countries, net price may depend on the indication but without full correlation to value.

## References

[1] Roche experimenting with new pricing models in oncology The Pink Sheet 10 June 2013.

[2] Bach P (2014). Indication-specific pricing for cancer drugs. JAMA.

[3] Kitamura M, Koch J Bloomberg 2016, November 16.

[4] Anno Fricke (2016). AMNOG soll auf Prüfstand. Ärzte Zeitung.

[5] German Social Code Book Five (SGB V), Section 35a.

[6] German Social Code Book Five (SGB V), Section 35a English summary.

[7] Transparency Commission Task http://www.has-sante.fr/portail/jcms/c_412210/en/commission-de-la-transparence.

[8] Transparency Commission Task English Summary http://www.has-sante.fr/portail/jcms/c_1729421/en/transparency-committee.

[9] HAS Information on Proper use contracts.

[10] Code de Santé Publique Article R5132-3.

[11] NSM Decision on Avastin temporary usage recommendation.

[12] AIFA Updated list of Records and Therapeutic web based Plans.

[13] AIFA CTS Decision 366.

[14] AIFA Pricing and Reimbursement http://www.agenziafarmaco.gov.it/en/content/pricing-and-reimbursement.

[15] Esposito L, Paganelli F (2012). Improving the quality of data in the Onco-AIFA register as a prerequisite for outcome research studies. Eur J Hosp Pharm.

[16] Navarria A, Drago V, Gozzo L, Longo L, Mansueto S, Pignataro G (2015). Do the current performance-based schemes in Italy really work? “Success Fee”: A novel measure for cost-containment of drug expenditure. Value Health.

[17] Spanish Official Bulletin, 25 July 2015.

[18] Interoperability of electronic prescriptions and advances in 12 regions.

[19] Brizuela L Estudio de implantación de receta electrónica en España. Farmacéuticos Comunitarios. 2014; 6(Suplemento1). http://www.farmaceuticoscomunitarios.org/journal-article/estudio-implantacion-receta-electronica-espana.

[20] National Institute for Health and Clinical Excellence (2008). Guide to the methods of technology appraisal.

[21] The Pharmaceutical Price Regulation Scheme 2014 Department of Health.

[22] National Cancer Drugs Fund List https://www.england.nhs.uk/wp-content/uploads/2015/09/ncdf-list-sept15.pdf.

[23] National Cancer Intelligence Network http://www.chemodataset.nhs.uk/home.

[24] NHS RightCare http://www.rightcare.nhs.uk/.

[25] The Swedish Pharmaceutical Reimbursement System English summary.

[26] Tandvårds- och läkemedelsförmånsverket (TLV) Types of reimbursement.

[27] National Pharmaceutical StrategyAction Plan 2014 http://www.government.se/contentassets/57e68c08f2f04e0490fb5638aac230f1/national-pharmaceutical-strategy---action-plan-2014.

[28] Tandvårds- och läkemedelsförmånsverket (TLV) Mission statement http://www.tlv.se/lakemedel/Utvecklingvardebaserad-prissattning/.

